# To Replay, Perchance to Consolidate

**DOI:** 10.1371/journal.pbio.1002285

**Published:** 2015-10-23

**Authors:** Lisa Genzel, Edwin M. Robertson

**Affiliations:** 1 Centre for Cognitive and Neural Systems, University of Edinburgh, Edinburgh, United Kingdom; 2 Institute of Neuroscience and Psychology, University of Glasgow, Glasgow, United Kingdom

## Abstract

After a memory is formed, it continues to be processed by the brain. These “off-line” processes consolidate the memory, leading to its enhancement and to changes in memory circuits. Potentially, these memory changes are driven by off-line replay of the pattern of neuronal activity present when the memory was being formed. A new study by Dhaksin Ramanathan and colleagues, published in *PLOS Biology*, demonstrates that replay occurs predominately after the acquisition of a new motor skill and that it is related to changes in memory performance and to the subsequent changes in memory circuits. Together, these observations reveal the importance of neuronal replay in the consolidation of novel motor skills.

## Abstract 2

This Primer examines the implications of a new study showing that the pattern of neuronal activity during the formation of a memory is replayed during sleep, changing memory circuits and enhancing the memory. Read the Research Article.

Transforming your first floundering attempts at a new skill into the smooth, powerful, graceful performance of an athlete requires practice. Perhaps 10,000 hours of practice [1]. Yet, practice, no matter how dedicated, is not sufficient. To be retained, our memories for a motor skill need to be consolidated [2]. A new study by Dhaksin Ramanathan and colleagues, published in *PLOS Biology* [3], provides powerful new insights into the mechanism responsible for motor skill consolidation.

## Memory Consolidation

**Fig 1:**
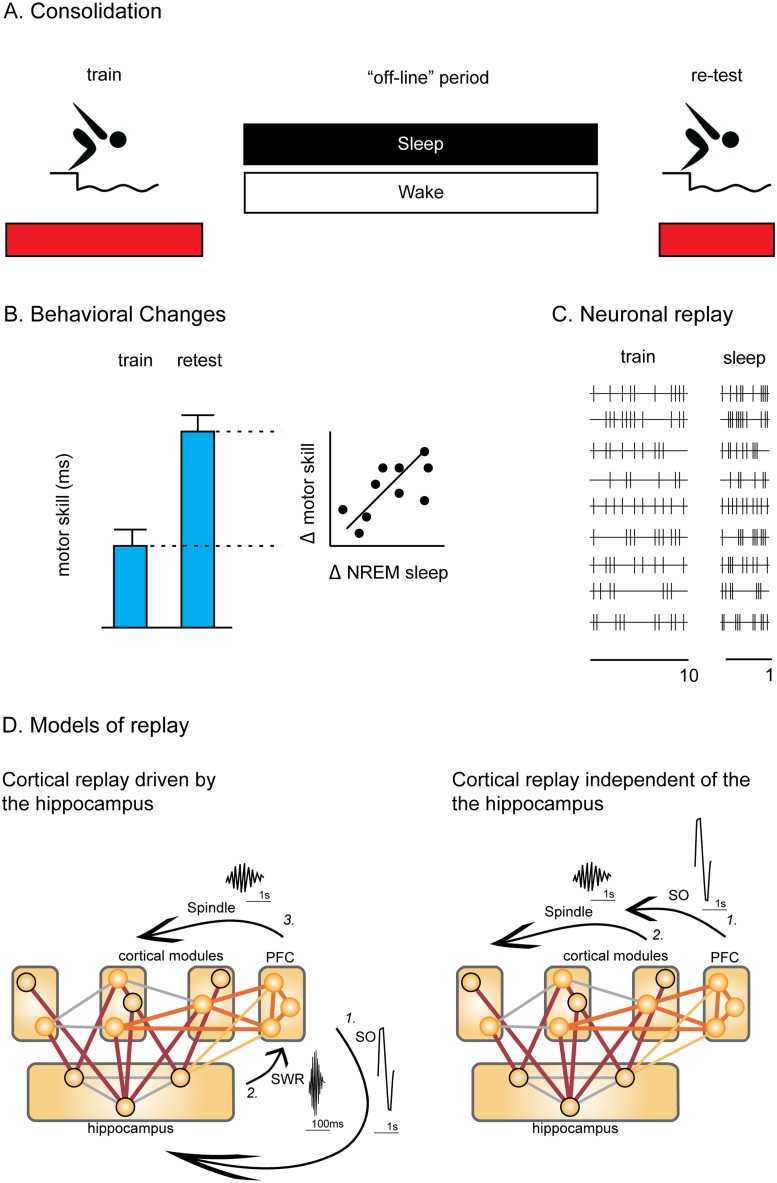
Neuronal replay and memory consolidation. **(A)** Following their acquisition at training, memories for motor skills continue to be processed off-line during wake or sleep. **(B)** These off-line process consolidate a memory, leading to motor skill improvements developing over sleep, which are often correlated with changes in non-rapid-eye-movement (NREM) sleep (for example, spindles; see Box 1). **(C)** Patterns of neural activity observed during learning are replayed seven to ten times faster off-line during sleep. **(D)** Cortical replay may be due to hippocampal replay **(left)**: (1) replay is initiated by a slow oscillation (SO) starting in the prefrontal cortex that travels to the hippocampus, where it is followed (2) by a sharp-wave ripple (SWR) that is accompanied by hippocampal replay. Subsequently, prefrontal cortical replay is initiated via the hippocampal-prefrontal cortex pathway (light orange), and transmitted (3) to other cortical areas, i.e., the motor cortex during sleep spindles. Alternatively, cortical replay may take place independently of the hippocampus **(right)**: (1) A SO occurs and is followed (2) by spindle for local cortical replay and processing. Overall replay is thought to strengthen within-cortical networks (lines in cortex in grey and orange) of memory encoding modules (circled in orange). Non-relevant information is not consolidated within the cortex (circled in black).

After its acquisition, a new motor skill memory continues to be processed “off-line” during consolidation (Fig 1A) [4]. A wide variety of changes can happen to a memory during consolidation. Frequently, consolidation is considered the stabilization of a memory, when it is made resistant to interference either from other memories or disruptive experimental techniques such as brain stimulation or protein synthesis inhibitors (for a review, see [5]). A motor skill may be enhanced during consolidation with a 20%–30% increase in performance between testing and retesting 6–12 hours later (Fig 1B) [6]. A memory may also be reorganized during consolidation, allowing people to gain insight into underlying patterns or structures within a mathematical puzzle or sequence of events, for example [7]. The circuits supporting a memory may also be reorganized during consolidation; for example, a memory may be reliant on a brain area, such as the hippocampus, before but not after consolidation (for an example, see [8]). Thus, consolidation can lead to a diverse set of changes, from the enhancement of a memory to a reorganization of the circuits critical to a memory; yet what remains poorly understood is how these off-line changes occur.

**Box 1. Sleep Oscillations**

During non-rapid-eye-movement (NREM) sleep, characteristic oscillations across a wide range of frequencies are seen. Different roles in memory consolidation processes have been proposed for different oscillations. (1) The slow oscillation (SO, 0.5–1 Hz) is seen throughout all NREM sleep and is visible in the surface electroencephalogram (EEG) as a K-complex. SO represent generalized up and down states, with generalized in- and decreases in firing rate of most neurons respectively. (2) Slow wave activity (SWA, delta waves, 0.5-4Hz) is characteristic of deep NREM sleep (slow wave sleep, SWS). (3) Cortical sleep spindles (13–16 Hz) with their characteristic waxing and waning form are hallmark of light NREM sleep, but also occur in deep NREM sleep. (4) In contrast to the above-mentioned oscillations, which can be seen in the surface EEG, the sharp wave ripple (SWR, 100–200 Hz) can only be measured with in-depth electrodes in the hippocampus.

## Neuronal Replay

Over 40 years ago, David Marr proposed the idea that neurons activated during practice are reactivated off-line as the basis for memory consolidation [9]. Consistent with this idea, many laboratories have found that the pattern of a neuron’s activity present during practice is similar, and at times even identical, to that same neuron’s pattern of off-line activity. Essentially, the pattern of activity during practice is “replayed” (Fig 1C). For some animals, such as songbirds, there is a very good match between the pattern of activity during practice and the subsequent off-line activity. However, for other animals, such as rodents, the match is less immediately clear because the pattern of neuronal activity is replayed over a much shorter time interval than during earlier practice (Fig 1C). Regardless, the pattern of a neuron’s activity during training is replayed off-line, and such neuronal replay has, over the last couple of decades, become one, if not the leading, contender as a mechanism responsible for memory consolidation.

## Links between Replay and Consolidation

**Fig 2:**
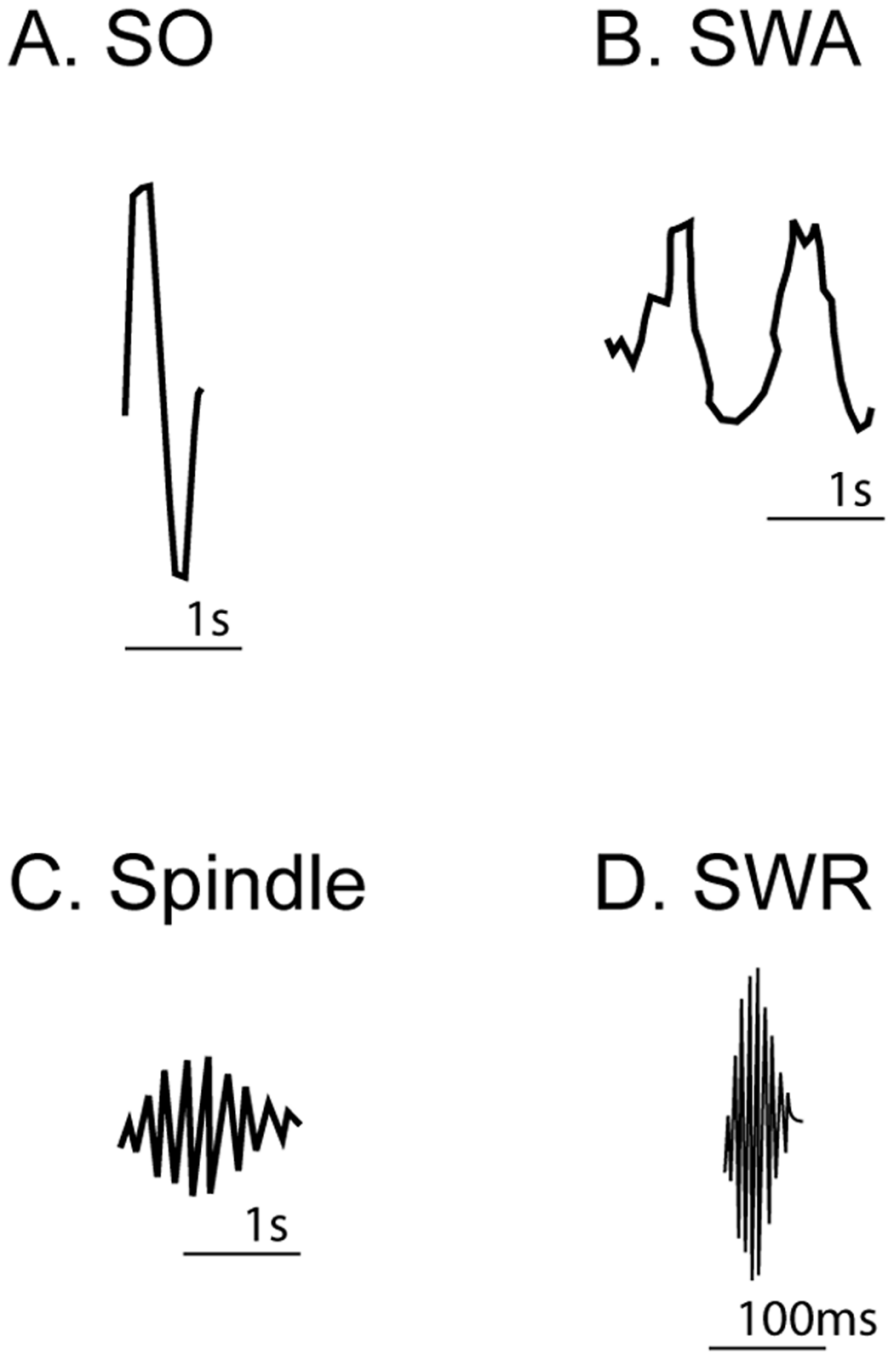
Oscillations of NREM sleep. **(A)** Slow oscillation (SO) present throughout NREM sleep; **(B)** slow wave activity (SWA) characteristic of deep NREM sleep; **(C**) sleep spindles characteristic of light NREM sleep; and **(D)** sharp wave ripple (SWR) associated with the hippocampus and memory processing. For details, please see Box 1.

Many studies have suggested an important link between neuronal replay and memory consolidation. Almost all replay has been observed during non-rapid-eye-movement sleep (NREM; see Box 1 and Fig 2), and consolidation-related changes—for example, enhancement in motor skill—are frequently related to NREM (for example, see [10]). Yet, the consolidation of some memories, such as those associated with fear, has been linked not with NREM but with rapid-eye-movement (REM) sleep (for a review, please see [11]). There have been a few reports of replay occurring during REM [12], although this is not as widely recognized as replay during NREM [13]. The amount and frequency of replay can be modified by the same factors that can modify consolidation, such as reward [14,15]. Sensory cues, such as tones played during sleep, can modify neuronal replay, and, in humans, similar cues alter both the replay within learning circuits during sleep and the consolidation of memories [16,17]. Finally, replay occurs in brain areas that are critical for learning, such as the hippocampus and the motor and parietal cortices [13,18,19]. Thus, mounting converging evidence has suggested an important connection between neuronal replay and consolidation.

## Challenges

Several important challenges remain in establishing a connection between consolidation and replay. There is no work demonstrating that memory consolidation is critically dependent specifically upon neuronal replay. Some studies have disrupted brain areas, in which neuronal replay is suspected or known to occur, and so prevented consolidation [20–22]. Others have disrupted sharp-wave ripples (SWR), which are frequently temporally associated with neuronal replay, also preventing consolidation [23]. Yet, the targeted disruption of neuronal replay and its effect upon subsequent memory consolidation has so far not been explored.

Another challenge to the link between neuronal replay and consolidation comes from the tasks that are used. Typically, due to technical constraints, studies investigating replay do not record neuronal activity after the learning of novel memory tasks that are expected to trigger subsequent memory consolidation. Instead, replay is usually reported after a well-learned task, such as simple maze navigation in rodents, which has likely already been consolidated. In this scenario, replay is being observed when little or no consolidation may be occurring. However, a study has shown replay following learning of new goals in a spatial memory task, and it showed that those replay episodes are correlated with subsequent memory performance [24]. Consistent with novel learning affecting replay, another study reported an increase in SWRs, which are thought to be associated with replay, after learning a new task [25]. Overall, replay may be a ubiquitous feature of off-line activity, following the performance of many tasks, regardless of their novelty. After all, replay may be important for off-line processes other than consolidation; for instance, following their retrieval, established memories are reconsolidated. Yet, the occurrence of replay may be affected by novelty, allowing it to play a central role in off-line processing of novel memory tasks and in their consolidation.

## Meeting the Challenges

Using a behavioral task combined with different manipulations (sleep deprivation, added sleep, added training), along with electrophysiological recordings, Ramanathan and colleagues show that neuronal replay in the motor cortex underlies the consolidation of a newly learned skill [3]. This study provides a number of important observations; it shows that (a) the development of off-line motor skill improvements in rats follows the acquisition of a novel motor skill, (b) these improvements are sleep-dependent, (c) neuronal replay is elicited when learning these novel motor tasks, (d) the replay is related to both the subsequent consolidation of the motor skill, and (e) the subsequent changes in the firing properties of motor cortical neurons. Together, these observations show that replay during sleep is important for the neurophysiological and behavioral changes that take place during consolidation. This work connects replay to consolidation in a convincing way.

## New Insights: Timing of Spindles and Replay

The authors used multiple techniques to measure neural replay in the motor cortex during sleep (principle component analysis, template matching, and binning) and showed many similarities, but also some differences, to replay in other cortical areas. Earlier work has shown that replay in the prefrontal cortex occurred immediately before sleep spindles, whereas, in the current work, motor cortical replay occurs further along into the spindle ([18] cf. [3]). This difference in the relative timing of replay and spindle may provide important clues about the processing and progress of replay across the cortex (Fig 1D).

A slow-oscillation (SO; see Box 1) is thought to start in the prefrontal cortex and travel to the hippocampus. A sharp wave ripple (SWR; see Box 1) follows the SO accompanied by hippocampal replay. Subsequently, there is a response of prefrontal cortex cells (~30–50 ms post SWR). When cortical spindles follow SWRs, a second larger wave of increased firing of cells and replay events can be observed in the cortex [18,26]. The replay occurs just before the spindle at the prefrontal cortex. By contrast, the onset of replay may be slightly delayed in the motor cortex, because it is being driven from the prefrontal cortex, and, as a consequence, replay is observed to occur at almost the same time as the spindle. The prefrontal cortex may be the route through which hippocampal replay is able to affect the motor cortex following motor skill learning.

## Is Motor Replay Hippocampal-Led?

Potentially, the motor cortical replay observed in the current study may be a late stage processing of hippocampal-led replay; alternatively, it may have occurred without hippocampal involvement (Fig 1D). Distinguishing between these possibilities is not yet possible because the current work focused exclusively upon examining replay in the motor cortex. However, it is at least conceivable that the motor cortical replay may have been hippocampal-dependent. Learning sequences of movements, such as those acquired by the rats in the current study, has been shown in humans to be dependent on the hippocampus (Box 2) [27–29]. The connectivity between the hippocampus and medial prefrontal cortex predicts overnight memory improvement in these tasks [30]. Overall, the hippocampus may be playing a role in motor cortical replay and motor skill consolidation. The beauty of the current work is, in part, that by using a motor sequence task, it opens the door to examining the importance of hippocampal replay in motor skill consolidation and, more generally, to the importance of this brain area to motor skill learning.

**Box 2. Different Types of Memory**

Traditionally, memories have been classified as being either declarative or procedural [31]. Declarative memories are those for particular events, episodes, or facts and frequently can be acquired quickly, perhaps over only a few repetitions. By contrast, procedural memories are associated with performance of a new skill or action and are acquired over many repetitions. The appeal of this classification, at least in part, is that these different types of memory are associated with distinct independent brain circuits. Declarative memories are dependent upon a circuit that includes the hippocampus and prefrontal cortex; whereas procedural memories, and particularly motor skill memories, are dependent upon a circuit that includes the motor cortex, striatum, and cerebellum [31]. Yet, declarative memory tasks, such as learning a list of words, disrupt the consolidation of a variety of motor skill memories [32–35]. An interaction between declarative and motor skill memories may be explained by an overlap in the circuits supporting the formation of these different types of memory. Learning and subsequently consolidating a motor skill memory leads to activity changes within brain areas that include the hippocampus, which have traditionally been considered part of the declarative memory system [28,36–38]. The hippocampus may be involved regardless of whether participants are or are not aware of the motor sequence (i.e., for both explicit and implicit learning) [28]. Earlier work in patients showed that the medial temporal lobe, which includes the hippocampus, is critical for some forms of motor skill learning [27]. Overall, declarative and procedural information is processed within hippocampal circuits. Memory circuits may not be segregated upon the basis of the type of information being processed (i.e., declarative versus procedural). Instead, different computations may be performed within different circuits; for example, the hippocampus may support temporal processing of information regardless of whether it is declarative or procedural.

## Replay and Brain State

Replay does not occur exclusively during sleep; however, its influence beyond the hippocampus to cortical areas may only occur during sleep. The present study found neuronal replay in the motor cortex associated with neurophysiological features such as spindles and slow oscillations, which only occur during sleep [3]. Similarly, another study reported prefrontal cortex replay only during episodes containing high delta power, which are indicative of sleep [18]. By contrast, hippocampal replay can be observed in quiet rest as well as sleep [13]. Perhaps only in sleep, when global network connections are favorable [39], can hippocampal-led replay reach the cortex or can pure cortical replay occur [40]. Thus, replay perhaps changes from being local to being global depending upon brain state (quiet rest versus sleep). This may affect the function of replay and could explain why the properties of consolidation differ over sleep and quiet rest [33,41] (for a review, see [6]).

## A Tale of Two Mechanisms

In addition to replay, another suggested mechanism for consolidation during sleep is “downscaling”—per Tononi and Cirelli, “with sleep homeostatically but non-specifically regulating synaptic weights to improve the signal-to-noise ratio of memory traces” [42]. The combined “push–pull” action of replay, on the one hand, and downscaling, on the other, may act in a complementary manner to aid the construction and updating of memory networks in the cortex (“push” equals potentiating important traces, whereas “pull” equals weakening irrelevant traces) [40,43,44]. For example, subsequent downscaling to increase the signal-to-noise ratio may augment the initial replay. However, whether or not downscaling plays an important role in the consolidation of the motor task used by Ramanathan et al. remains an open question. Downscaling has been associated with the consolidation of some motor skills [45]. The properties of a memory task may determine the mechanisms that are engaged for its consolidation. For example, a sequential component to a task may elicit a strong replay effect, while other tasks, with little or no sequential component, may be predominately consolidated by downscaling. In sum, these two mechanisms may have complementary roles across a diverse array of memory tasks, with the extent, nature, and timing of their contribution being determined by the properties of the task being consolidated.

Overall, the present work has provided important insights into the relationship between replay and consolidation. Replay is greater when a motor skill is novel and requires consolidation, whereas replay is diminished as a motor skill becomes well learned and is in less need of consolidation. The new study also links replay to the change in the properties of the motor memory circuits. Together, these results deepen our understanding of the connection between replay and consolidation. Perhaps just as importantly, this study brings new questions into sharper focus. It remains poorly understood how replay causes those widespread but specific changes in functional connectivity that are associated with consolidation. Demonstrating, as this study does, the connection between replay and changes in the property of motor memory circuits is a first important step along the path to answering these critical questions.

## Abbreviations

EEG: electroencephalogram
NREM: non-rapid eye movement
REM: rapid eye movement
SO: slow oscillation
SWA: Slow wave activity
SWR: sharp-wave ripple
